# Two cases of respiratory infection due to *Pasteurella multocida*: Acute and chronic form

**DOI:** 10.1002/rcr2.1388

**Published:** 2024-05-28

**Authors:** Hironoshin Onizuka, Akihiro Hara, Kotaro Shiratori, Akito Yamamoto, Toshihiro Shirai

**Affiliations:** ^1^ Department of Respiratory Medicine Shizuoka General Hospital Shizuoka Japan

**Keywords:** acute respiratory distress syndrome, nodular shadow, *Pasteurella multocida*

## Abstract

We present two cases of *Pasteurella multocida* (*P. multocida*) respiratory infection. The first case involves a 62‐year‐old female with abnormal chest shadows, a history of bronchial asthma, and colorectal cancer. Endobronchial ultrasound with a guide sheath (EBUS‐GS) revealed granulomatous changes, and *P. multocida* was cultured. The second case is a 64‐year‐old female presenting to the emergency department with progressively worsening chest pain and dyspnea, with *P. multocida* detected from her sputum culture. Treatment with penicillin antibiotics resulted in symptom improvement and normalization of CT findings. These cases indicate the importance of considering *P. multocida* in respiratory infections, given the patients' history of pet ownership and the nonspecific imaging findings and symptoms. This highlights the necessity for accurate diagnosis and appropriate antibiotic treatment, particularly in cases where animal traumatic exposure is not detected.

## Graphical abstract

We present two cases of respiratory infection caused by *P. multocida*, effectively managed with penicillin antibiotics, illustrating both chronic and acute courses. These cases indicate the importance of considering *P. multocida* in respiratory infections, given the patients' history of pet ownership and the nonspecific imaging findings and symptoms. This highlights the necessity for accurate diagnosis and appropriate antibiotic treatment, particularly in cases where animal traumatic exposure is not detected.

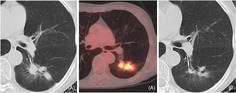

## INTRODUCTION

*Pasteurella multocida* (*P. multocida*) is an aerobic, Gram‐negative short rod bacterium that commonly resides in the upper respiratory tract and digestive tract of mammals.^1^ It has been reported that nearly 100% of cats and 75% of dogs are carriers of *P. multocida*.^1^ In humans, infections with *P. multocida* often appear as localized infections at sites of animal‐related abrasions or bites, leading to cellulitis and abscess formation.^2^ Additionally, non‐traumatic infections transmitted from animals, particularly respiratory infections, have been documented. Non‐traumatic infections with *P. multocida* suggests the possibility of droplet transmission.^3^ In some cases, these infections can result in chronic infection or sepsis shock.^1^

## CASE REPORT

### Case1

**FIGURE 1:**
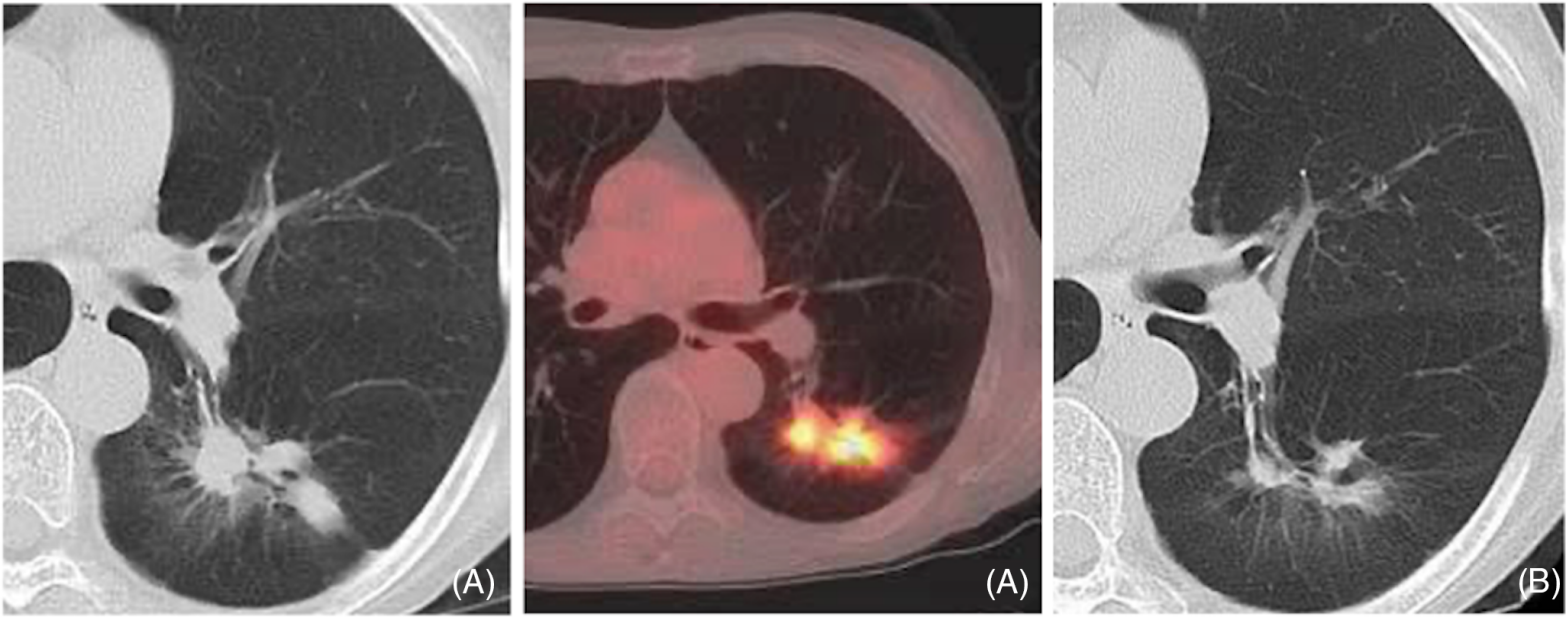
(A) CT scan and PET‐CT scan when she first came to our hospital, and (B) CT scan 2 months later after she started AMPC/CVA.

A 62‐year‐old female presented to our hospital following an incidental finding of abnormal chest shadows during a routine examination. Two months earlier, a chest x‐ray revealed these anomalies in the left middle lung field, prompting subsequent chest computed tomography (CT) imaging at a nearby clinic. The CT scan demonstrated multiple lung nodules, the largest measuring 18 mm in diameter in the left lung S6 region (Figure 1A). She was referred to our facility for further assessment. The patient presented sputum production and had a medical history of bronchial asthma and colorectal cancer. A former smoker of 37 years, with a consumption rate of 20 cigarettes per day. She was currently on medications including tiotropium bromide hydrate, budesonide‐formoterol fumarate hydrate, montelukast, and theophylline. She had a dog for 10 years and denied any recent travel or avian exposure. On examination, vital signs were within normal limits, and she appeared well‐oriented with no signs of anaemia or jaundice. There were no palpable cervical lymph nodes, and cardiac and pulmonary auscultation revealed normal findings. Abdominal and neurological examinations were unremarkable. Laboratory investigations showed a white blood cell count of 10,500/μL with differential percentages indicating a neutrophil predominance. Additional parameters including biochemical markers, and tumour markers were within normal ranges and microbiological tests were negative for common pathogens. PET‐CT imaging revealed an accumulation in the left lung S6 area with an SUVmax of 8.25 (Figure 1A). Subsequent endobronchial ultrasound with a guide sheath (EBUS‐GS) revealed granulomatous changes, with *P. multocida* isolated from bronchoalveolar lavage (BAL) fluid. Acid‐fast bacilli culture was negative. Amoxicillin/clavulanic acid (AMPC/CVA) at a dosage of 750 mg per day was administered for a duration of 2 months as a therapeutic regimen for lung abscess. A follow‐up CT scan 2 months later demonstrated resolution of the previously observed abnormalities (Figure 1B), correlating with symptomatic improvement.

### Case2

A 64‐year‐old female presented to the emergency department with worsening chest pain and dyspnea over the preceding day. She had a history of type 2 diabetes mellitus complicated by diabetic nephropathy, neurogenic bladder, and prior cerebral infarction. Dependent on welfare assistance, she owned a pet dog. Her medication included physostigmine, idrapril, torasemide, rosuvastatin, ezetimibe, calicivirinase, pregabalin, cilostazol, furosemide, amlodipine, febuxostat, trichlorthiazide, esomeprazole, insulin degludec, liraglutide, and insulin aspart. Vital signs showed bradycardia with a heart rate of 45 bpm, hypotension at 89/54 mmHg, hypothermia measuring 28.9°C, tachypnea with a rate of 16 breaths/min, and hypoxemia with oxygen saturation at 93% on 5 L/min supplemental oxygen. The Glasgow Coma Scale was E3V4M4. Additionally, bilateral coarse crackles and peripheral coldness were observed.

**FIGURE 2:**
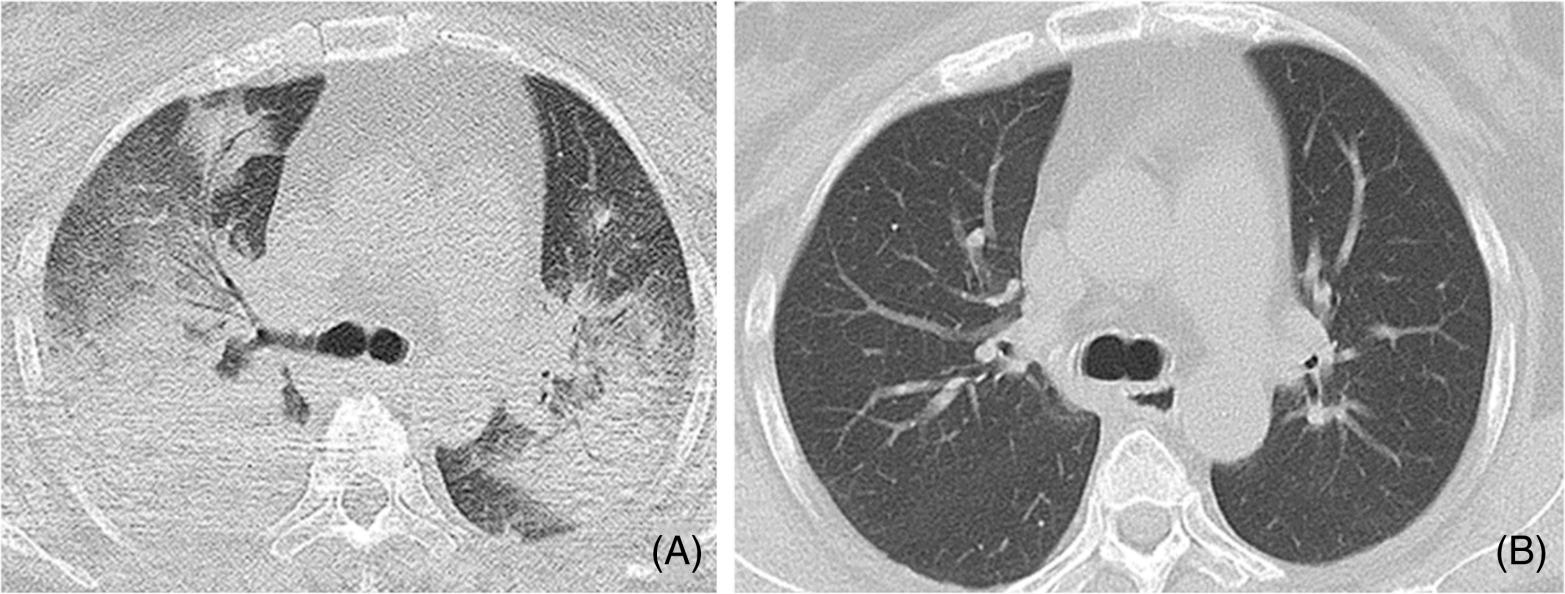
A CT scan (A) when she first came to our hospital, and (B) 4 months later after she started AMPC/SBT.

Laboratory investigations showed a white blood cell count of 5500/μL, elevated blood urea nitrogen at 68 mg/dL, creatinine levels of 2.46 mg/dL, increased liver enzyme levels including AST at 685 U/L, ALT at 363 U/L, and LDH at 949 U/L. Hyperglycemia was noted with glucose levels at 201 mg/dL, indicative of poorly controlled diabetes with an HbA1c of 7.2%. Furthermore, CRP levels were elevated at 7.6 mg/dL. CT imaging demonstrated diffuse bilateral infiltrates, as depicted in Figure 2A. Her blood gas showed PaO2 at 75.8 torr and PaCO2 at 65.6 torr. She was diagnosed acute respiratory distress syndrome and treatment comprised endotracheal intubation, hydrocortisone, sivelestat, and broad‐spectrum antibiotics (meropenem 1.5 g/day, levofloxacin 250 mg/day) for 5 days. Extubation occurred on day two, followed by nasal high flow therapy initiation. *P. multocida* was isolated from sputum culture on day five, prompting de‐escalation to ampicillin/sulbactam 9 g/day for 2 weeks. The patient recovered, with a subsequent CT scan (Figure 2B) 4 months post‐discharge demonstrating resolution of abnormalities. *P. multocida* was also detected from her dog and she was instructed to stop the contact with her dog.

## DISCUSSION

We presented two cases of respiratory infection caused by *P. multocida*, effectively managed with penicillin antibiotics, illustrating both chronic and acute courses. Clinical manifestations of *P. multocida* respiratory infections are nonspecific, pneumonia, bronchitis, and lung abscess.^3^ Risk factors include animal contact, advanced age, immunodeficiency, liver cirrhosis, smoking, and chronic pulmonary disorders.^3^, ^4^ In our cases, one patient had smoking history, while the other had type 2 diabetes mellitus, suggesting they have predispositions. CT scans in *P. multocida* infections reveal nonspecific features such as infiltrative shadows, centrilobular nodules, or bronchiectasis.^3^, ^4^ Differential diagnoses may include cancer or abscess, as seen in our cases. Diagnosis was confirmed through BAL and sputum culture. Initial antibiotic therapy for *P. multocida* infections typically choose penicillin.^3^ Alternatives like levofloxacin or doxycycline are considered in cases of resistance or allergy.^3^ Given reports of beta‐lactamase production in *P. multocida* infections from bites, susceptibility testing is imperative.^5^ Although healing generally takes 2–4 weeks, our cases demonstrated resolution within 4 months, highlighting the importance of prolonged treatment, particularly in patients with underlying lung diseases. Regular symptom monitoring and follow‐up CT imaging are essential for assessing treatment efficacy and ensuring resolution.

If there are any respiratory symptoms, *P. multocida* should be considered in patients with pet ownership history or animal contact history. Diagnosis confirmation via BAL or sputum culture is crucial. Penicillin or doxycycline is the treatment cornerstone for *P. multocida* respiratory infections. Regular symptom monitoring and follow‐up CT imaging are essential for assessing treatment efficacy and ensuring resolution. To prevent reinfection, it is necessary to instruct patients to avoid excessive contact with pets.

## CONFLICT OF INTEREST STATEMENT

None declared.

## ETHICS STATEMENT

The authors declare that appropriate written informed consent was obtained for the publication of this manuscript and accompanying images.

## Data Availability

The data that support the findings of this study are available on request from the corresponding author. The data are not publicly available due to privacy or ethical restrictions.
